# Complete Genome Sequence of Lactobacillus nenjiangensis SH-Y15, Isolated from Sauerkraut

**DOI:** 10.1128/MRA.01473-19

**Published:** 2020-06-04

**Authors:** Jiale He, Yan Liu, Fengzi Li, Hao Wu, Hongyan Yang

**Affiliations:** a Key Laboratory of Saline-Alkali Vegetation Ecology Restoration, Ministry of Education, College of Life Sciences, Northeast Forestry University, Harbin, China; University of Maryland School of Medicine

## Abstract

We isolated a strain of Lactobacillus nenjiangensis named SH-Y15 from traditional suan-cai used in northeastern China because it has a high capacity for degrading nitrites at low temperatures. The complete genome of SH-Y15 contains a single circular chromosome and a plasmid. The complete length is 2,249,893 bp, and the G+C content is 39.68%.

## ANNOUNCEMENT

Nitrite widely exists in fermented foods and is more prevalent in vegetable products. Upon entering the body, high levels of nitrite can pose a range of health risks (1). Lactic acid bacteria are common microorganisms in naturally fermented foods and are generally considered to be safe probiotics (2). Related studies have shown that lactic acid bacteria can degrade nitrite during growth and metabolism, thus ensuring food safety (3); therefore, it has become a trend to isolate lactic acid bacteria from natural fermentation systems for further study (4).

Lactobacillus nenjiangensis is a kind of lactic acid bacteria that is sometimes isolated from suan-cai (5). Few studies have been conducted to determine its functions in fermented vegetable systems, because its genomic background is unclear. We isolated L. nenjiangensis from traditional suan-cai. The process used is briefly described as follows. A sample of fermented sauerkraut juice was subjected to plate dilution and cultivation in modified MRS medium (6) (glucose was substituted for xylose) for 48 h at 30°C. After being streaked twice, one colony was inoculated into modified MRS broth. After cultivation for 48 h (optical density at 600 nm [OD_600_], 2.0), 3 ml of the culture was centrifuged at 5,000 × *g* for 2 min. The pellet was used for extraction of genomic DNA using a bacterial DNA extraction kit (D3350-01; Omega, USA). The 16S rRNA gene of the strain was amplified, and the PCR product was sequenced. The sequence was further confirmed after the GenBank alignment initially confirmed that it had ≥99% similarity to other L. nenjiangensis strains.

Illumina and PacBio single-molecule real-time (SMRT) libraries were sequenced using the Illumina HiSeq X Ten and PacBio Sequel platforms, respectively, according to the instruction manual for each platform. Sequencing libraries were prepared with an AxyPrep Mag PCR purification kit (AP-PCR-50G; Axygen, Union City, CA, USA), and genomes were sequenced using the 2 × 150-bp paired-end protocol for the HiSeq X Ten system (Illumina, San Diego, CA, USA) (7). Read data sets were trimmed to improve quality with the software package Trimmomatic (version 0.39) (8). Our Illumina sequencing generated 4,024,912 raw reads and 2,684,616 clean reads for a sequencing depth of 178×. The PacBio library was prepared by using the SMRTbell template preparation kit version 1.0 (100-259-100; Pacific Biosciences, Menlo Park, CA, USA) to generate a large-fragment library of genomic DNA (9). A total of 47,962 reads were obtained after PacBio sequencing for a sequencing depth of 307×. (10).

Proovread (version 2.12) software (11) was used to evaluate the quality of both PacBio and Illumina reads, and preliminary assembly was performed using Falcon (version 0.3.0) (12) with default settings (default parameters were used for all software unless otherwise noted). GLIMMER (version 3.02) software (13) was used to predict genes on the basis of the assembly results, and the open reading frames and gene length distribution of each strain genome were obtained. RNAmmer (version 1.2) software (14) was used to predict rRNAs, and tRNAscan-SE (version 1.3.1) software (15) was used to predict tRNA regions and tRNA secondary structures. The whole genome of L. nenjiangensis SH-Y15 consists of 54 tRNA genes and 15 rRNA genes. The extent of genome coverage obtained was 100%. Finally, based on the analysis of the sequencing samples, Circos software (version 0.64) (16) was used to obtain the genome circle map and the plasmid circle map.

### Data availability.

The chromosomal and plasmid sequences of Lactobacillus nenjiangensis SH-Y15 have been deposited in GenBank. The accession numbers of the chromosomal and plasmid sequences are CP043939 and CP043940, respectively. The SRA numbers for the reads are SRR10446101 and SRR10446102, respectively.
